# Revisiting the mitogenetic effect of ultra-weak photon emission

**DOI:** 10.3389/fphys.2015.00241

**Published:** 2015-09-07

**Authors:** Ilya Volodyaev, Lev V. Beloussov

**Affiliations:** Laboratory of Developmental Biophysics, Department of Embryology, Faculty of Biology, Moscow State UniversityMoscow, Russia

**Keywords:** methods to detect MGE, mitogenetic methods, mitogenetic effect, distant interaction, non-chemical signaling, ultraweak photon emission, spontaneous chemiluminescence

## Abstract

This paper reviews the 90 years long controversial history of the so-called “mitogenetic radiation,” the first case of non-chemical distant interactions, reported by Gurwitsch (1923). It was soon described as ultraweak UV, emitted by a number of biological systems, and stimulating mitosis in “competent” (in this sense) cells. In the following 20 years this phenomenon attracted enormous interest of the scientific community, and gave rise to more than 700 publications around the world. Yet, this wave of research vanished after several ostensibly disproving works in late 1930-s, and was not resumed later, regardless of quite serious grounds for that. The authors discuss separately two aspects of the problem: (1) do living organisms emit ultraweak radiation in the UV range (irrespective of whether it has any biological role), and (2) are there any real effects of this ultraweak photon emission (UPE) upon cell division and/or other biological functions? Analysis of the available data permits to conclude, that UV fraction of UPE should be regarded real, while its biological effects are difficult to reproduce. This causes a paradox. A number of presently known qualities of UPE were initially discovered (predicted?) by the “early workers” on the basis of biological effects. Yet the qualities they discovered were proved later (the UV component of UPE, the sources of UPE among biological systems, etc…), while the biological effect they used for UPE “detection” remains questionable. Importance of this area for basic biology and medicine, and potential usefulness of UPE as a non-invasive research method, invite scientists to attack this problem again, applying powerful research facilities of modern science. Yet, because of complexity and uncertainty of the problem, further progress in this area demands comprehensive examination of both positive and negative works, with particular attention to their methodical details.

## Introduction

Investigation of both ultraweak photon emission (UPE) and non-chemical distant interactions (NCDI) in living mater was started in 1920-s by a well-known Russian histologist Alexander Gurwitsch (1874–1954). His research was an attempt to answer a question, not responded in its full scale even now: “what are the causes of cell division?” Combining several observations, Gurwitsch concluded that this event required a coincidence of two factors: (1) **internal** cell “preparedness” to division, and (2) **external** impulse, i.e., a signal coming from the outside and “switching on” the (already prepared) mitosis. He suggested, that the external impulse was non-chemical (i.e., a kind of radiation), and induced “collective excitation” of special molecular receptors located on the cell surface. [Mark that the notion of membrane receptors became widely used only several decades later. See original works (Gurwitsch, 1911, 1923), and their discussion in Bateman (1935); Gurwitsch (1988); Van Wijk (2014).

To test the hypothesis of the “non-chemical external impulse,” Gurwitsch performed his famous “onion root experiment” (Gurwitsch, 1923). Two onion roots as even and smooth as possible were located perpendicular to each other and mutually centered, so that the tip of root No1 (acting as the “emitter” of the “impulse”) was directed toward the division zone of root No2 (acting as the “recipient”). The authors made histological sections of the “recipient” root, and calculated the number of mitotic figures in the exposed and non-exposed halves of the root. The exposed side possessed significantly higher proportion of cells in mitosis than the non-exposed side (see more in Section MGE on Plant Meristem). This phenomenon was called “mitogenetic effect” (MGE).

MGE was also detected, if a quartz plate was fixed between the two roots, and was not detected, if the roots were separated with glass or nontransparent materials (Gurwitsch, 1924; Reiter and Gabor, 1928a). Chemical isolation of the roots did not affect the results. Based on these and other data (see more in Section Physical Qualities of Mitogenetic Radiation), the acting factor was concluded to be UV light of very low intensity, and was called “mitogenetic radiation.”

The phenomenon was soon shown widely spread in the living nature. A summary of possible inductors and recipients, as well as the conditions necessary for observing MGE, will be given in Section Definitions. Some of the most important critical works on this subject will be reviewed in Sections MGE on Plant Meristem and MGE on Yeast and Bacteria. Although MGE appeared quite capricious, once a stable effect was obtained, it could be used as a standard “detector” of mitogenetic radiation. The latter was soon found a sensitive and absolutely non-invasive marker of the physiological state of its emitter. A number of laboratories (Profs. Gurwitsch, Blacher and Pesochensky in the USSR, Prof. Siebert in Germany, Prof. Wolf in the Netherlands, Prof. Rahn in the USA) that obtained stable “basic MGE,” used it for further research and clinical diagnostics (see Section MGE as a Non-invasive Probe for Detecting Physiological and Pathological States of Cells and Tissues).

The mechanism of MGE became the central research question from the very beginning. The very first works on this problem already contained evidences, that the inducing factor was non-chemical, and “behaved” like UPE in UV spectral range (see Section Physical Qualities of Mitogenetic Radiation). This was soon followed by attempts to detect it with physical light-sensitive devices—photographic plates, modified Geiger counters, and finally PMTs (described in detail in Sections Photon Emission from MGE Inductors and partially in Newer Works on UPE). From early 60-s research on UPE from biological systems gradually spread around the world (briefly reviewed in Section Newer Works on UPE), and UPE became a well-established phenomenon.

Yet, it is not a triumph of MGE. Mitogenetic radiation (if existing) is by convention a signal, stimulating cell division. UPE is a side effect of radical oxidative processes with no biological roles, except a way to “get rid” of potentially dangerous energy surplus. Mitogenetic radiation should belong to UV range (190–240 nm). UPE is mostly visible. Yet, the first works on UPE originated from the problem of MGE, and a number of UPE qualities had been discovered in mitogenetic experiments. Can this be a coincidence? I.e., can the “early works” on UPE-MGE have been a big fallacy, that “predicted” UPE only extrinsically? Although this is the viewpoint of a number of influential authors (Zhuravlev, 1973; Quickenden and Tilbury, 1985; Vladimirov and Proskurnina, 2009), we consider it a personal opinion, based on belief, but not on evidences.

The main aspects of our defense of the problem of MGE are the following:

1. The total number of works on MGE is more than a thousand, including those in top rating journals (e.g., at least 10 articles in Nature), and those by well-known and respectable scientists [e.g., Profs. Rahn (USA), Wolf (Holland), Reiter, Gabor (Germany), Gurwitsch, Frank, Chariton, Pesochensky (USSR)].
2. In the vast majority of those works, MGE was detected. The number of works “disproving” MGE was less than 20. All the “disproving” works we could obtain, were done with principal deviations from the conditions, necessary to get MGE. We will separately discuss this point for each of them in Sections MGE on Plant Meristem, MGE on Yeast and Bacteria, Photon Emission from MGE Inductors, and Imitation of MGE with Artificial Sources of UPE. Thus, no work by this time ever refuted MGE as a phenomenon.
3. Presently, UV component of UPE can be regarded proven (Troitskii et al., 1961; Gurwitsch et al., 1965; Tilbury and Quickenden, 1988). It coincides with mitogenetic radiation in spectral range and the culture growth phase, when it is observed. The sources of the UV component are definitely different from those of visible UPE in biological systems. These, as well as some other facts will be discussed in Section Newer Works on UPE and Discussion.

Still, MGE is not well established either. The reasons for that will be discussed in detail in Section Discussion, but probably the main one is capriciousness and uncertainty of the effect. We consider it an intrinsic property of the phenomenon, which demand scrupulousness in details to obtain good reproducible results.

Thus, we reckon the problem of MGE still unsolved and undeservedly forgotten. If this phenomenon were finally shown real and credible, it would be an important breakthrough for the whole biological science, with a number of very serious applications (see Section MGE as a Non-invasive Probe for Detecting Physiological and Pathological States of Cells and Tissues). Otherwise, it should be univocally closed. We reckon that further progress in this area demands comprehensive examination of both positive and negative works, with the focus on methodical details and reproducibility.

## Mitogenetic effect. “biological detection”

### Definitions

In this section we give basic definitions of the mitogenetic effect, and summarize conditions necessary to obtain it.

**Mitogenetic effect (MGE)**—is a change in mitotic regime in a cell culture or tissue under external non-chemical influence of another biological object.

**Recipients of MGE** (often called “detectors”)—are cell cultures and tissues, capable of showing MGE under external influence.

**The known recipients are:**

- Bacterial and yeast cultures in lag phase (Wolf and Ras, 1931; Ferguson and Rahn, 1933; Tuthill and Rahn, 1933);
- “Aging” yeast cultures (Baron, 1926, 1930);
- Tissue cultures (Guillery, 1929);
- Plant meristem (Gurwitsch, 1923; Reiter and Gabor, 1928b; Siebert and Seffert, 1933);
- Eye cornea (cornea of frog, Gurwitsch and Anikin, 1928);
- Developing embryos [eggs of sea urchin (Magrou and Magrou, 1927); frog eggs (Reiter and Gabor, 1928b); eggs of Drosophila (Wolf and Ras, 1934)].

**Inductors of MGE**—are those objects that can produce MGE in proper recipients when put in proper conditions.

**The known inductors are:**

- Actively growing microbial (Magrou and Magrou, 1927; Siebert, 1928a; Baron, 1930; Acs, 1932) and tissue cultures (Gurwitsch and Gurwitsch, 1934);
- Working muscles and heart (Siebert, 1928b; Gurwitsch and Gurwitsch, 1934);
- Excited neurons (Gurwitsch, 1934);
- Blood of healthy people (Gurwitsch and Salkind, 1929; Siebert, 1930; Pesochensky, 1942);
- Malignant tumors (Gurwitsch and Gurwitsch, 1945);
- Resorbed and regenerating tissues (Blacher et al., 1932);
- Certain chemical reactions (Gurwitsch, 1968; Rahn, 1934a).

**Definite non-inductors are:**

- Not growing or slowly growing cultures (Gurwitsch and Gurwitsch, 1934; Rahn, 1936);
- Internal parts of the body (Rahn, 1936);
- Blood of cancer patients (Gurwitsch and Salkind, 1929; Siebert, 1930; Pesochensky, 1942);
- Blood of people with some other diseases (anemia, sepsis, pneumonia, scarlatina) (Protti, 1930; Siebert, 1930);
- Blood of old and exhausted people (Protti, 1930; Gurwitsch and Gurwitsch, 1934; Pesochensky, 1942).

### Methods of observing MGE

Selection of proper inductors and recipients still cannot guarantee the effect. A number of other conditions, necessary to obtain MGE were shown in different works. Here we summarize them in short.

#### The experimental setup

1. **Optical contact between the inductor and the recipient**.
  The optical channel should be transparent down to 190 nm.
  “Even quartz can be used only if it is of very high purity” (Hollaender, 1936).
2. **Distance between the inductor and the recipient**.
  Optimal distance is 1–10 mm (the smaller the better).
  The maximal “working” distance depends on the inductor, induction length and special conditions like “interrupted induction” (see below) (Gurwitsch, 1932, 1968; Gurwitsch and Gurwitsch, 1948).
3. **Induction length**.
  The effect is non-linearly dose-dependent, with clear suppression phase at high doses (Sussmanowitsch, 1928; Gurwitsch, 1932; Wolf and Ras, 1933).
  The length of induction should be optimized for every conditions a new, at least in the diapason 1–120 min.
  Examples of the optimal induction length:
  - 1–2 h (inductor, onion root; recipient, onion root) (Gurwitsch and Gurwitsch, 1934);
  - 30 min (inductor, yeast; recipient, yeast) (Tuthill and Rahn, 1933);
  - 15–30 min (inductor, bacteria; recipient, bacteria). 60 min induction gave no effect; 2 h induction and more gave depression (Wolf and Ras, 1933).
4. **“Interrupted induction” and spectral analysis**.
  The idea of this method was to check if the mitogenetic radiation had any special temporal order. For this sake, the inductor and the recipient were separated with a non-transparent disc, which had sectorial slits of various size and mutual position. The disc was rotating at constant speed, and thus the recipient was periodically exposed to the inductor (through the slits) or screened from it (with the rest of the disc). Mutual position of slits determined the temporal pattern of such “interrupted exposure” (i.e., periods of exposure and screening).
  For some inductors (malignant tumors) the value of MGE in this system depended only on the total duration of induction. For others (functioning nerves, muscles, etc…) the induction temporal pattern was crucial. The authors claimed, that in the second case periodic patterns (periods of exposure ~1 ms, periods of screening ~50 ms) gave MGE several times higher (!), than in standard induction; while more complicated patterns (the same rotation frequency, but irregular position of slits) removed the effect (Gurwitsch and Gurwitsch, 1934, 1959). See Section Can UPE Transfer Information? for discussion.
  In practical sense, this method allowed the authors to increase the distance between the inductor (of the second type) and the recipient, and even to set a monochromator in between. Thus, the method of “MGE spectral analysis” was invented (see Section Physical Qualities of Mitogenetic Radiation).
5. **Development of the effect**.
  MGE needs some time after the end of induction to become detectable (Gurwitsch and Gurwitsch, 1934; Rahn, 1936). This time is mostly 30 min–2 h (Tuthill and Rahn, 1933) if the measured parameter is budding index or percent of mitoses, and 1–4 h if it is the culture density (Ferguson and Rahn, 1933; Wolf and Ras, 1933). For yeast cultures this time could also be as short as 5–10 min, but if only the smallest buds were counted (the so called “fast method” used in Gurwitsch's lab since 1945) (Gurwitsch and Eremeev, 1947; Gurwitsch, 1968).

#### The recipient culture

1. **Physiological state**.
  MGE can be observed on either lag-phase (Ferguson and Rahn, 1933; Tuthill and Rahn, 1933; Wolf and Ras, 1933), or “aging” cultures, but is more pronounced on the first.
  Lag phase yeast recipients should be inoculated from post-diauxic^1^ phase, and better from agar, than from suspension cultures (Rahn, 1936).
  Lag-phase bacterial recipients should be inoculated from 2 to 4 days old inoculum cultures (Wolf and Ras, 1931, 1933; Ferguson and Rahn, 1933). “24 h <*E. coli*> cultures never reacted; cultures 48 h old or still older always responded” (Rahn, 1936).
  No effect was found on younger or older cultures (Tuthill and Rahn, 1933). I.e., neither actively growing (or soon after), nor stationary phase (G_0_) cultures were sensitive to mitogentic radiation.
2. **Period of sensitivity**.
  Every recipient has a “window of sensitivity” (or competence to MGE), which depends on the state of the inoculum culture. The deeper the inoculum culture has progressed in post-diauxic phase, the later its “sensitivity window” “opens.”
  Lag-phase yeast cultures plated from 24 h inoculum were sensitive within 0–1 h after plating. Cultures plated from 6 days inoculum—within the period 2–2.5 h after plating. Cultures plated from 10 days old inoculum were not sensitive to mitogentic stimuli at all (Tuthill and Rahn, 1933).
  Bacterial recipient cultures were sensitive either immediately after plating (2–4 days old inoculum, diluted in fresh medium to 20,000 cells/ml) (Wolf and Ras, 1933), or just before plating (2–4 days old inoculum exposed to the inductor, and immediately diluted in fresh medium to 50–5000 cells/ml) (Ferguson and Rahn, 1933).
3. **Culture density**.
  No effect was found on too dense cultures (Rahn, 1936). Too diluted cultures either didn't grow, or didn't show any sustainable effect (Gurwitsch and Gurwitsch, 1934).
  Recommended culture density:
  - 20,000 cells/ml (staphylococci, suspension culture) (Wolf and Ras, 1931);
  - <100,000 cells/ml (*E. coli*, suspension culture. Good results obtained at concentration 50 and 5000 cells/ml) (Ferguson and Rahn, 1933);
  - Single cells on agar medium, not forming groups (*S. cerevisiae*, agar culture). “Cells <should be> far enough apart not to influence each other” (Rahn, 1936, p. 68).
4. **Media composition**.
  The effect was inconsistent if the recipient was plated on “standard media.” To optimize the effect, Rahn used broth diluted 1:10 with water (*E. coli*, suspension cultures) (Ferguson and Rahn, 1933). Gurwitsch, on the contrary, found stable effects on oversaturated media (18 balling beer wort) (Gurwitsch and Gurwitsch, 1934).

#### Special precautions

1. Suspension cultures can be induced only in very thin layers, <0.5 mm (Wolf and Ras, 1933; Rahn, 1936). “Thicker layers of the medium absorb all radiation and take off the effect” (Rahn, 1936).
2. The radiation can be reflected by quartz or glass plates, used in the experimental setup.
3. Induction should be done at “diffuse daylight” (Potozky, 1930). MGE is not observed in complete darkness or at bright light (Gurwitsch and Gurwitsch, 1934).
4. Some inductors of MGE (e.g., yeast cultures) should stay at “diffuse daylight” for at least 2 h before the induction (Potozky, 1930).
5. Most of the experiments on MGE were performed in the presence of atmospheric oxygen, but without special saturation with either oxygen or other gases. A few attempts to get MGE in anaerobic conditions led to negative effects (Gurwitsch and Gurwitsch, 1934).
6. No external UV. MGE is not observed in the presence of external sources of (even weakest) UV (Ruyssen, 1933).

For more detailed examination of the demands to get MGE we recommend a painstaking review by Rahn (1936), and also (Gurwitsch, 1932; Hollaender, 1936).

### Experimental protocols

In the original “standard yeast protocols” by Gurwitsch and Baron, budding index (BI, % of budding cells) of the induced culture was compared to that in control. Although the cultures used were “aging,” according to the given experimental tables control BI was still ~20–30%. The induced cultures mostly showed a 40–80% increase of budding, relative to the control “background level.” E.g., ${BI}_{control}=30\%;\;{BI}_{induced}=45\%;MGE=\frac{45\%-30\%}{30\%}=50\%\;\left(relative\right).$ In “yeast protocols” by Rahn, control culture was in the lag-period, and thus had practically zero budding: *BI*_*control*_ ~ 0−5%. Comparing this to *BI*_*induced*_ ~ 20%, the authors obtained much more noticeable results.

Thus, in the following section, we give what we consider the best experimental protocols for MGE detection. The “bacterial protocol” is originally from Wolf, but optimized by Rahn; the “yeast protocol” was suggested by Rahn anew.

### MGE on bacterial cultures. The method by Ferguson and Rahn (1933)

**Culture:** *E. coli*. **Medium:** Broth, diluted 1:10 with sterile water. **Temperature:** 37°C.

**Recipient:** Culture of *E. coli* in 1:10 diluted broth, 2–4 days old (24 h old cultures don't work), grown at 37°C.

**Induction:** The recipient is placed in a quartz Petri dish in a very thin layer (~0.5 mm) and induced from the bottom. Immediately after the end of induction it is diluted with fresh medium to cell density 10^1^–10^4^ cells/ml (50 cells/ml and 5000 cells/ml work well; 500,000 doesn't work) and incubated at 37°C for 8 h. Cell concentration is measured every 2 h (the authors used plate count method).

In Wolf and Ras (1931, 1933) the recipient culture was first diluted till concentration 20,000 cells/ml, and then exposed to the inductor (in a layer ~0.5 mm).

A good inductor is a 4 h old (at 37°C) agar surface culture of *E. coli*. A Petri dish with such an inductor is placed under the recipient with no material separating it from the recipient, except the quartz bottom of the recipient Petri dish. For such an inductor optimal time of induction is 15–30 min (5 min and 60 min induction give no effect) (Rahn, 1936).

### MGE on yeast cultures. The method by Tuthill and Rahn (1933)

**Culture:** Burgundy yeast. **Medium:** Raisin extract (see Rahn, 1936, p. 68); raisin agar (1:2 diluted raisin extract, 3% agar). **Temperature:** 30°C.

**Inoculum:** Yeast suspension culture in raisin extract, 24 h old, is flooded over a Petri dish with sterile solid raisin agar. The dish is incubated for 24 h at 30°C and used to inoculate the recipient culture (it must be free of buds when used; otherwise an older culture should be taken).

**Recipient:** Cells from the culture above are washed off the dish with 5 ml of sterile water; then diluted with water 1:100, and used to flood dishes with solid raisin agar (the surplus of liquid is drained off at once). “The yeast cells are so far apart <on the agar surface> that the buds can be counted directly on the agar surface” (Rahn, 1936, p. 69).

The induction should start immediately (<30 min) after plating.

**Induction:** The (freshly prepared) recipient is covered with quartz plate and induced from the top. A good inductor is an exponential phase yeast culture. Optimal time of induction for it is 30 min.

**Measurement:** After the end of induction, the induced recipient is incubated at 30°C for several hours. Budding index (% of cells with buds) is detected (the best effect is observed 0.5–1.5 h after the end of exposure). To calculate the number of buds, the culture is fixed with “a cotton wad with tincture of iodine” (Rahn, 1936, p. 69), placed directly in the Petri dish. “Soon after that, a cover glass can be placed on the agar surface, and the slightly-stained yeast is observed *in situ*, eliminating all possibility of breaking off buds by smearing on glass” (Rahn, 1936, p. 69).

### MGE on plant meristem

The very first experiment on MGE was performed on onion roots. One of the roots (“inductor”) was located perpendicular to the other one (“recipient”), the tip of the “inductor” directed onto the “recipient” division zone and separated from it with a quartz plate (see Gurwitsch, 1923). The proportion of cells in mitosis (calculated from the number of mitotic figures) was found significantly higher on the exposed side. Outside the region of exposure the distribution of mitotic figures was uniform (Table 1).

**Table 1:** **Average number of mitoses on cross sections (10 μm thick) of the exposed and the opposite side of the root (quoted from Gurwitsch, 1988)**.

	**% of cells in mitosis**		
	**Exposed side**	**Opposite side**	**Difference**
Outside of the range of exposure	54.25 ± 16.77	54 ± 16.99	0.25 ± 3.97
Exposed part	65.60 ± 8.38	47.50 ± 7.45	17.83 ± 6.91
Outside of the range of exposure	42.86 ± 8.60	43.05 ± 8.75	−0.18±3.16

Such experiments were repeated in other laboratories with positive (Reiter and Gabor, 1928a; Siebert, 1928a; Loos, 1930) or negative results (Rossmann, 1928; Moissejewa, 1929; Taylor and Harvey, 1931).

#### Critical works

**Figure 1:**
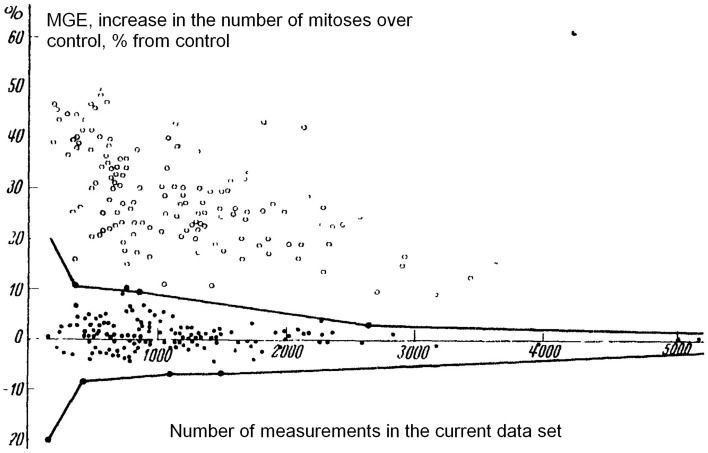
**Graphical summary of all results on MGE by Gurwitsch et al. in 1923–1929 (both inductor and recipient—onion roots, from Schwemmle, 1929)**. Each point represents a set of data. Horizontal axis, number of mitoses counted in the work; vertical axis, increase over control, %. Black dots, controls (no MGE); circles, experiments (regarded as positive MGE). Black line, limit lines for control (lines including the whole control distribution. Do not mix them with standard error or standard deviation). All circles (i.e., experiments with positive MGE) are outside the limit lines of the control distribution.

In a well-known critical work (Taylor and Harvey, 1931) positive results by other authors were suggested artifacts coming from natural non-uniform distribution of mitoses (The authors got fluctuations in control roots of ~50%). Still, in works by Reiter and Gabor fluctuations in control were ~20% (125 experiments), and in works by Gurwitsch et al. 10% (several hundred experiments). Both groups obtained significant results. An independent statistical analysis of all their data by 1929, was performed in Schwemmle (1929), and showed their statistical significance (see Figure 1).

The critique by Moissejewa (Moissejewa, 1929, 1931) consisted mainly of the following statements: (1) she couldn't obtain any repeatable results; (2) mechanical stress of one side of the root stimulated mitoses in it; (3) illumination of roots during the experiment setting could cause their phototrophic curving. The author also assumed that successive MGE workers took the best roots for experiments, and worse roots for controls, and also selected only those microtome sections, that conformed the expected results. Thus, she accused the groups of Gurwitsch, Reiter and Gabor and others of deliberate falsification, which is certainly the worse offense for any scientist. Unfortunately, this way of thinking is typical for those who were unable to obtain positive results themselves.

A methodically irreproachable work on onion root was later published by Paul (1933), which considered all criticism, and excluded any artifacts. The work gave doubtless positive results, but was strangely ignored in later critical reviews (Bateman, 1935). In our opinion, this work gave the final significant answer to the problem of MGE in plants [See (Rahn, 1934b, 1936) vs. (Hollaender and Schoeffel, 1931; Hollaender, 1936) for more details].

### MGE on yeast and bacteria

In further research MGE was also shown on yeast (Baron, 1926) and bacterial cultures (Wolf and Ras, 1931). The effect was repeated in Acs (1932), Frank (1932), Tuthill and Rahn (1933), and was not in Richards and Taylor (1932), Hollaender and Claus (1937). The total literature on MGE on these objects includes no less than 500 publications [See (Gurwitsch, 1932, 1968; Rahn, 1936; Gurwitsch and Gurwitsch, 1948) vs. (Bateman, 1935; Hollaender and Claus, 1935; Hollaender, 1936) for reviews and discussion].

#### Critical works

Another well-known work from Taylor (Richards and Taylor, 1932), performed on yeast, was done very carefully, but missed important methodical details. In particular (see Section Methods of Observing MGE for more details and quotation):

1. Suspension cultures were used.
2. Nothing is said about the culture physiological state, and according to their experimental tables, it was exponential phase.
  *MGE is observed only in lag-phase or “aging” cultures. The authors quoted this statement, but preferred to violate it*.
3. The medium was optimal for yeast growth.
4. The temperature was 28°C.
  *MGE is mostly observed in suboptimal conditions, including lower temperature and poor or oversaturated media*.
5. The recipient was put in flasks of 1.5–2 ml, and the flasks were fixed in a big container with the inductor suspension.
  *MGE on suspension cultures is observed only in very thin recipient layers*.
6. The induction lasted 2–24 h.
  *Yeast cultures show good MGE only at induction less than 2 h; longer induction gives suppression*.
7. The culture density was measured right after the induction.
  *The induction could affect the culture density no earlier than 1–2 h after its end*.
8. Nothing is said about the quartz quality.

Thus, it was impossible to observe MGE in this work from nearly any of the conditions shown above.

Another influential work by this group (Taylor and Harvey, 1931) was devoted to physical registration of mitogenetic emission. The authors used photographic plate, and obtained no results. This is also natural, as photographic plates are inappropriate tools to detect UPE (see more in Section Detection with Physical Devices).

##### Hollaender and claus

The most crucial work for the whole topic of MGE is the one by Hollaender and Claus (1937). It is a 100 pages manuscript, with a lot of raw tables and painstaking details. The main impression when reading it is unsurpassable difficulty of even slight attempt to try this field again. The authors were working in rubber gloves, with grounded quartz plates (they even grounded themselves), did 8 passages of every culture before it was used, and had many other precautions like that. Still, their work shows a lot of principal problems. Here we summaries its methodical details (see Section Methods of Observing MGE):

1. The main objects were *E. coli* and *Serratia marcescens*.
2. The cultures were grown on agar; but the induction and further growth were done in suspensions.
3. The experiment was set as follows: (1) agar cultures 15–19 h old (they later shifted to cultures 39 h old) were washed off the agar (2) with an inorganic salt solution, (3) immediately exposed to the inductor, diluted in the culture medium and incubated at 32°C. Samples were taken from the exposed culture after “repeated rapid twirling.”
  1. *For E. coli, only cultures 48 h or older (at 37*°*C) were proved sensitive to MGE. Anyway, the cultures become competent to MGE several hours after the end of active growth (see Section Methods of Observing MGE). Here the growth curves are not given, and the culture age was selected from irrelevant motives (“lowest number of double cells, highest percentage of live cells and ease of removal from agar” for 15 h cultures, and ability to “perform experiments of large size…without loosing…a well-defined lag-phase” for 39 h cultures, Hollaender and Claus, 1937)*.
  2. *The “inorganic salt solution” used as the culture medium during the exposure time, consisted of NaCl, KCl, CaCl*_2_*, and water. No other worker tried such a specific medium for induction*.
  3. *Recipient cultures have certain periods of sensitivity to the induction. Thus, 24 h yeast cultures are sensitive immediately after plating on the new medium, and 6-day cultures—2 h later. No MGE can be seen if the recipient is induced outside this period. Here this was not checked at all*.
4. Induction was done (1) in small cups 2 cm in diameter and 1–2 cm high at 37°C (2) with constant stirring of the recipient, and (3) lasted from 5 s to 12 min.
  1. *Suspension cultures should be induced in very thin layers (*<*0.5 mm). No effect can be obtained at thicker suspensions*.
  2. “*Constant stirring” and “rapid twirling” of the recipient culture are very specific conditions. No other worker ever tried MGE under them*.
  3. *Since the conditions were unknown, the induction length should have been tried at least up to 2 h. In “positive” works it was always carefully optimized*.
5. The recipient cultures had concentration mostly 1.5–2 × 10^5^ cells/ml.
  *In Wolf and Ras (1933) it was* 2 × 10^4^ *cells/ml; in Ferguson and Rahn (1933) the recipient culture was diluted to 50*–*5000 cells/ml; no effect was obtained on more dense cultures*.
6. The work was done under very low light (“in a room without windows …[with] a 25-watt globe, contained in a dark green or dark brown bottle.”
  *This is a good way to standardize the light conditions, which were rather unclear from Gurwitsch's publications. Still, in most “positive” works it was definitely lighter in the room, with only specific precautions against artificial UV*.

Thus, most of the conditions were new, and never checked before. The authors took a lot of doubtful precautions, but didn't optimize the conditions of primary importance (the culture age, the induction start and duration, the medium content, the light conditions). Besides, some of them were definitely against previous recommendations (the culture age, the thickness of the recipient layer).

##### Others

Critical works were also published by Nakaidzumi and Schreiber (1931), Kreuchen and Bateman (1932) and Westenberg (1935).

In all of them principal deviations from the methods recommended in “positive works” were made.

- In Nakaidzumi and Schreiber (1931) yeast cultures 9–12 h old (at 25°C) were used as recipient. This corresponds to exponential phase, which was long known incompetent to show MGE (see Section Methods of Observing MGE).
- In Kreuchen and Bateman (1932) the recipient culture was taken in too high concentrations (at which no MGE can be obtained either), and the intensity of (artificial) mitogentic inductor was ~10^4^ times higher than recommended in Chariton et al. (1930) (see Section Can UPE Transfer Information?).
- Unfortunately we didn't have a chance to see the works of Westenberg in original. Still, they were much less influential, and their methodic was criticized in detail in a number of works (see Gurwitsch and Gurwitsch, 1948).

#### New works

The works on MGE mostly stopped in 1940-s, except several groups in the USSR (Konev et al., 1963, 1966; Gurwitsch, 1968) and some groups continuing studies on cancer diagnostics. Pesochensky defended a Dr. Sci. dissertation “The phenomenon of <MGE> quenching at cancer and pre-cancer diseases” (Pesochensky, 1942) in Leningrad in 1942, during the Siege. Gurwitsch was evacuated from Leningrad in 1941, and became the head of a new institute in Moscow. Yet, he was assailed by Lysenko during his company vs. genetics etc…and although remained at large, was devoid of his lab and any opportunity for research work. He was several times nominated for the Nobel Prize (“Nomination Database: Alexander Gurwitsch”), and awarded the Stalin prize in 1941.

The topic of nonchemical interactions was “revisited” later by a number of authors, mostly in the USSR. A more than 20 years research was performed by Kaznacheev et al. in 1960–1980-s. The authors showed that cytopatic effect induced in a cell culture by viruses or toxic chemicals, could be “transferred” to another (recipient) cell culture, chemically separated but optically coupled with the first one. A huge amount of work by this group, including seasonal changes in the effect, analysis of reproducibility etc…, was summarized in Kaznacheev and Mikhailova (1981). Similar works were performed by Kirkin (1981), and later by Nikolaev (Nikolaev, 2000; Beloussov et al., 2007), Burlakov (Burlakov et al., 2000), Beloussov (Beloussov et al., 1997, 2000), Trushin (2004) and others (we apologize to those not mentioned) (For recent reviews see, Trushin, 2003; Cifra et al., 2011; Scholkmann et al., 2013).

##### Quickenden

In 1970-s–1990-s a serious set of works connected to MGE, was published by Quickenden et al. The authors detected significant photon emission from growing yeast cultures, in both visible and UV spectral range (see below), but couldn't obtain biological MGE (Quickenden and Tilbury, 1985). Here we summarize their technical details (see Section Methods of Observing MGE for more details and quotation):

1. They used diploid laboratory strains of *S. cerevisiae*.
2. The inductor culture was in exponential phase.
3. The recipient culture was (1) in stationary (G_0_) phase (10 days-old in a rich growth medium at 28°C and oxygen saturation) or (2) in lag-phase—just after seeding the former (G_0_) culture in fresh medium.
  *It was clearly shown in “early works,” that no MGE was observed in G*_0_*. The culture should be either post-diauxic, or plated from such one, with the induction start depending on its age*.
4. The cultures used, were (1) suspension, (2) bubbled with oxygen and (3) at 28°C, which is all much better physiologically, but totally different from the conditions of early works.
5. The induction was done in “test tubes” of 10 ml volume.
  *MGE in suspension cultures is observed only in very thin layers*.
6. The induction started immediately after (or immediately before) plating the recipient in fresh medium.
  *The induction start is one of principal parameters for MGE observation, and should be optimized for the culture age used. Besides, the older the inoculum culture, the later the induction should start. Here the recipient culture is much older than any one used before, and the induction start is the earliest possible*.
7. The only time of induction tried here was 30 min.
  *It should be optimized at least in the diapason 1*–*120 min for each new conditions*.

Thus, the conditions used in these works were totally new, and never checked for MGE before. None of principally important parameters of the experiment were optimized (or even checked for the effect), and a number of conditions were not applicable for MGE at all (the method of induction, and probably the culture age).

##### Others

A work by Wainwright et al. (1997) was also done under absolutely new conditions, but the authors were lucky to obtain a good effect. Unfortunately, they could not make it reproducible (probably because of the new and not optimized conditions). The same can be said about the works by Musumeci et al. (Grasso et al., 1991).

## MGE as a non-invasive probe for detecting physiological and pathological states of cells and tissues

Contrary to natural expectations, only a small part of numerous studies performed in several labs headed by Alexander Gurwitsch and later by his daughter, Prof. Anna Gurwitsch, was devoted to the study of the “basic MGE.” In most cases, the effect was used as a refined tool for non-invasive and immediate detection of a large number of physiological and biochemical processes taking place in normal and pathologically modified cells and tissues (Worth mentioning, most of these labs were affiliated to medical bodies). To make this possible, spectral analysis of MGE was widely used, and its modification, the so called selective scattering of external UV by biological and chemical samples, was elaborated (Gurwitsch and Gurwitsch, 1947).

The reported results can be reviewed here only in broad outlines. A substantial bulk of investigations was dealing with neural excitation and brain tissue activity (Gurvich, 1937; Gurwitsch and Gurwitsch, 1959). The authors showed propagation of MGE activity along the excited nerve fiber going with the rate of electric impulse (Gurwitsch, 1934; Gurvich, 1937). They also reported that MGE spectra of nerves depended on the nature of exciting agents. In another series of experiments flashes of photon emission (called “degradational radiation”) were detected immediately after application of stressful agents (see Gurwitsch and Gurwitsch, 1945).

In all these cases spectral analysis of MGE revealed a number of fast and as a rule reversible reactions (undetectable by standard physiological and biochemical methods). The authors related these reactions to formation and/or destruction of what they called “non-equilibrium molecular constellations” (i.e., excited supermolecular associations, Gurwitsch and Gurwitsch, 1948). Thus, they previewed the existence and the biological role of activated metastable complexes, their delocalized electron-excited states (e.g., in photosynthesis) and other phenomena called dissipative structures.

Applications of such “MGE-research” to the problem of malignant growth is of an excessive interest. It was first discovered that tumors were very active MGE inductors (Gurwitsch and Gurwitsch, 1945). At the same time, blood of cancer patients (contrary to that of healthy people) stopped emitting mitogenetic radiation at the earliest stages of malignization, long before any histological signs (Siebert, 1930; Pesochensky, 1942, 1947). The authors attributed this phenomenon to secretion of a specific protein, which they called “cancer quencher.” This discovery was successfully used for early diagnosis of cancer diseases in 1930–1940-s not only in the USSR (Gurwitsch and Salkind, 1929; Pesochensky, 1942, 1947), but also in Germany (Siebert, 1930). The reported statistics of the coincidences between data from such “MGE-diagnostics” and standard diagnostic methods is impressive (see Pesochensky, 1947). However, later this method was forgotten. Yet, it is quite obvious that cancer pathology is such an important problem that none of its details, whether they are of practical purpose today or not, should be missed.

## Detection with physical devices

### Physical qualities of mitogenetic radiation

From the very first works on MGE, physical qualities of its mediator were among central problems of research. According to (Gurwitsch, 1924; Reiter and Gabor, 1928b; Siebert and Seffert, 1933), they are identical to those of extremely weak UV:

1. MGE can be obtained only in direct vision of the inductor;
2. The MGE-inducing factor can be reflected with UV reflecting mirrors;
3. The MGE-inducing factor can pass through quartz (of high purity) or very thin layers of glass or water (25 μm);
4. It cannot pass through thick glass plates, gelatin, or any non-transparent materials.

In further investigations “spectra” of MGE were obtained by separating the inductor and a set of recipients with a prism (Reiter and Gabor, 1928a) or a monochromator (Frank, 1929; Kannegiesser, 1931). Spectra published by Gurwitsch's school belong to the area 190–250 nm, those by Reiter and Gabor—330–340 nm (For discussion of spectral properties see, Gurwitsch and Gurwitsch, 1934; Hollaender, 1936; Rahn, 1936).

### Photon emission from MGE inductors

The first attempts to measure “mitogenetic radiation” with physical devices, were made with the use of photographic plates (Reiter and Gabor, 1928b; Taylor and Harvey, 1931), and later with photoelectric chambers (Chariton et al., 1930; Schreiber and Friedrich, 1930). The results were either negative (Taylor and Harvey, 1931) or inconsistent (Reiter and Gabor, 1928b).

A new type of photo sensitive technique, suggested by Rajewsky (1931), and soon reproduced by Frank (Frank and Rodionow, 1931), and others (Lorentz, 1929; Taylor and Harvey, 1931), was based on modified Geiger-Muller counters. Such a counter had a quartz window and a special photosensitive layer spread over the cathode inside the counter tube. Light quanta that got through the quartz window and hit the cathode, gave rise to a discharge in the counter, and were thus detected. These devices were constructed manually, and demanded sophisticated adjustment, with no standard procedures or criteria. Naturally, they had very different sensitivity, and even approaches to estimate it.

In Frank and Rodionow (1931), Rajewsky (1931), Siebert and Seffert (1933), UPE from different MGE inductors was detected successfully. Its intensity was 10–10^3^ quanta/cm^2^/s. In Lorentz (1929), Schreiber and Friedrich (1930), Taylor and Harvey (1931), Kreuchen and Bateman (1932), Seyfert (1932), Grey and Ouellet (1933), Hollaender and Claus (1937) the results were negative. Most of these authors claimed their devices to have very high sensitivity, and meant their results to be a disproval of MGE. Yet, the best, and methodically perfect works on this topic, done by Barth (1934), Grebe et al. (1938), and Audubert (1939), gave the final answer to this problem:

1. UPE from many biological objects was shown (blood of healthy people, growing yeast and bacterial cultures, tumors, etc.);
2. Its intensity estimate was the same as in Frank and Rodionow (1931), Rajewsky (1931)—10–10^3^ quanta/cm^2^/s;
3. It was either detected in UV, or had a UV component;
4. It correlated with biological MGE, i.e., “active” MGE inductors gave UPE, and “passive” did not.

In Barth (1934) previous failures to detect UPE were discussed in detail, and principal technical problems and artifacts were outlined. In particular:

1. Wrong position of the cathode, which produced interference from electrostatic fields (Lorentz, 1929);
2. Small size of the cathode (Kreuchen and Bateman, 1932), or its wrong position (Seyfert, 1932) which gave insufficient angle of light collection, and low signal / noise ratio;
3. Loss of sensitivity of the photosensitive layer (Lorentz, 1929);
4. Too high leakage resistance, which led to electrical breakups, and noise increase (Kreuchen and Bateman, 1932);
5. Usage of non-emitting objects (Lorentz, 1929; Grey and Ouellet, 1933).

[See (Bateman, 1935; Hollaender and Claus, 1935) vs. (Barth, 1934; Rahn, 1936; Audubert, 1939; Gurwitsch and Gurwitsch, 1948) for more discussion].

### Newer works on UPE

It is probably no need and even impossible to summarize here the present-day situation around UPE from living objects. The very fact of it is well-established, and its generally accepted mechanism is oxidative free radical processes with mostly lipid substrates (Boveris et al., 1981; Popp et al., 1988; Vladimirov and Proskurnina, 2009; Cifra and Pospíšil, 2014).

Standard and irrefutable registration of UPE became possible after the photomultiplier tubes (PMT) were invented (1930-s–1940-s), and developed to their maximum efficiency (1940-s–1950-s). The first generally known publications on UPE were done on plants by Colli and Facchini (1954), and on animal tissues by the group of Tarusov (Tarusov et al., 1961a,b). Unfortunately, the 1930-s works on Geiger-Muller counters described above, are usually not remembered in this respect, although the first credible detections are surely belonging to them.

#### Tarusov's group

Another regrettable thing is that the absolute majority of publications by Tarusov and coworkers from 1960-s to 1970-s were published in Russian and are inaccessible for English-reading researchers even as citations [See (Slawinska and Slawinski, 1983; Vladimirov and Proskurnina, 2009; Voeikov, 2010) for a minimal list of those publications].

The authors showed:

1. UPE from animal (Vladimirov and Litvin, 1959; Tarusov et al., 1961a) and plant (Vladimirov and Litvin, 1959) tissues;
2. Its fermentative and non-fermentative mechanisms (Popov and Tarusov, 1963; Zhuravlev, 1973);
3. Chemically active compounds (reactive oxygen species);
4. Main substrate (membrane lipids);
5. Spectrum of UPE (the presently known spectrum of ROS recombination).

For more detail see reviews (Vladimirov, 1966; Tarusov et al, 1967). Present day reviews can be seen in Vladimirov and Proskurnina (2009), Voeikov (2010), Cifra and Pospíšil (2014), Pospíšil (2014).

#### UV component

The UV component of UPE was also shown in a number of works (Troitskii et al., 1961; Gurwitsch et al., 1965). In an extensive work (Konev et al., 1966) the group of Konev detected UPE from several dozens of various species, ranging from bacteria to vertebrates and higher plants. The mechanism of the UV emission was not discovered in detail, but it was shown different from lipid peroxidation, and supposedly connected to protein synthesis (Konev et al., 1963).

**Figure 2:**
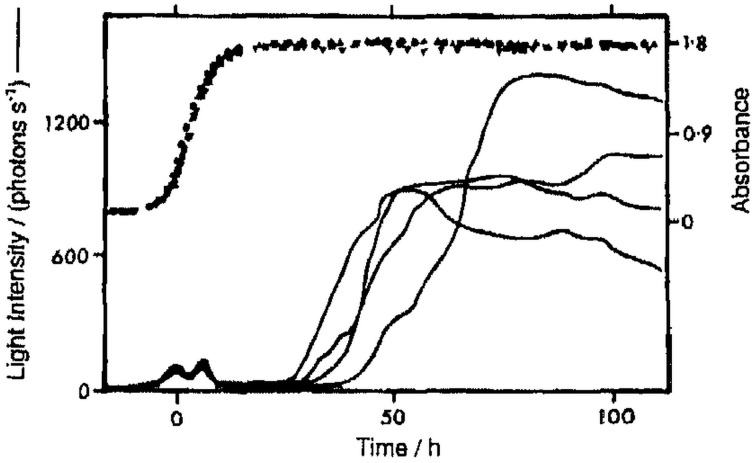
**Growth and UPE curves of oxygenated suspension cultures of ***C. utilis*** at 33°C**. Time given relative to the point of half-maximum growth (from Tilbury and Quickenden, 1992). Two distinctly different phases of UPE are present: (1) UPE from exponential phase cultures (two small peaks around 0 h) and (2) UPE of post-diauxic or stationary phase cultures 40–110 h).

**Figure 3:**
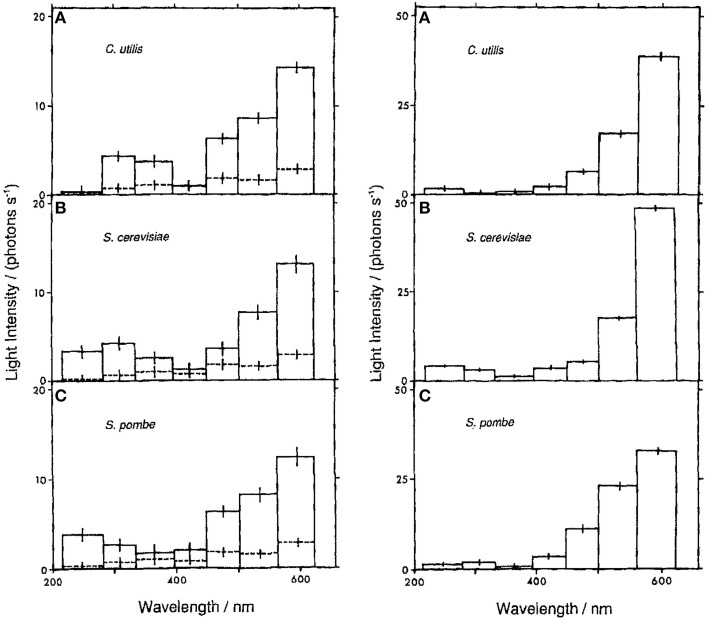
**UPE spectra of oxygenated suspension cultures of ***C. utilis*** (A), ***S. cerevisiae*** (B), ***S. pombe*** (C) at 33°C (solid lines)**. Left, growing (exponential phase) cultures; right, stationary phase cultures. Dotted lines, UPE of the medium (from Tilbury and Quickenden, 1992). Spectra of stationary phase UPE (right column) correspond to ROS recombination (Tarusov et al, 1967; Boveris et al., 1981). Spectra of exponential phase UPE (left column) have two components: (1) visible (corresponds to ROS recombination), and (2) UV (unknown mechanism, corresponds to UPE spectra shown in Troitskii et al. (1961), Gurwitsch et al. (1965), and to spectra of MGE (Frank, 1929).

Later, in a vast series of works, Quickenden et al. tried to verify phenomena of both MGE and UPE. The first attempt failed due to low sensitivity of the photo-measuring device (Metcalf and Quickenden, 1967), but later the authors managed to detect UPE from growing yeast cultures (Quickenden and Que Hee, 1974). In further works by this group the following facts were demonstrated:

1. All microorganism cultures tested [*S. cerevisiae* (Quickenden and Que Hee, 1974, 1976; Quickenden and Tilbury, 1983, 1991), *S. pombe* (Quickenden et al., 1985), *C. utilis* (Tilbury and Quickenden, 1992), *E. coli* (Tilbury and Quickenden, 1988)] possess growth-dependent UPE.
2. The UPE has two distinct phases (see Figure 2):
  - UPE of growing cultures (*start*, exponential phase; *max*, around half-maximum density; *duration*, ~1 day; the peak has 1 or 2 distinct maxima).
  - UPE of stationary cultures (*start*, post-diauxic, or stationary phase (2–8 days old); *duration*, several days or more; variable dynamics).
3. Total intensity of UPE is:
  - Growing cultures, 10–10^2^ quanta/cm^2^/s;
  - Stationary cultures, 10^2^–10^3^ quanta/cm^2^/s.
4. UPE has broad spectra at least from 200 to 600 nm with definite UV component for growing cultures (Figure 3).
  - Growing cultures, 20–40% UPE in UV;
  - Stationary cultures, <10% UPE in UV, or no UV component.
5. The sources of UPE are:
  - Visible components, lipid peroxidation, excited oxygen dimer;
  - UV component, unidentified. Not lipid peroxidation; not cosmic-rays excited fluorescence; not major biochemical reactions or protein synthesis. Oxygen dependent. The authors underline that “ultraviolet emissions are of similar intensity and wavelength to those designated as mitogenetic radiation by Gurwitsch.” They also suggest “oxidative side reactions associated with protein synthesis” as a possible source of this emission (Tilbury and Quickenden, 1992).
6. All detected UPE is oxygen-dependent (there is no UPE from anaerobic cultures).
7. In respiratory deficient cultures:
  - Visible UPE is 5–10 times higher than in normal strain;
  - UV UPE is equal or even two times lower than in normal strains.

The last two facts cannot be well explained now, but might help to find the source of the UV component in future.

## Can UPE transfer information?

Thus, by this time UPE from living systems is a well-established fact. However, whether it can be connected to the mitogenetic effect, i.e., whether UPE can physically transmit (any) signal in real conditions, remains an open question.

There is a number of woks published during the whole period since 1920-s with various considerations *pro* and *contra* feasibility of UPE-based signaling. Here we adduce the most important of them.

### Gurwitsch's scheme

Gurwitsch gave the following scheme of the process:

1. Mitogenetic radiation is a UV photon emission with intensity of 10–10^3^ photon/s.
  *UPE of exactly this intensity is presently well established, but its UV component is mostly doubted (Cifra and Pospíšil, 2014). Yet, we consider the works by the groups of Konev and Quickenden a definite proof of its existence (see Section Newer Works on UPE)*.
2. Mitogenetic radiation originates from recombination of free radicals.
  *This hypothesis was based on theoretical considerations and “mitogenetic spectra” (Gurwitsch and Gurwitsch, 1934, 1959)*.
  *It is well proven now, but only for visible UPE. Yet there are no established mechanisms for generation of UV quanta in biological systems (Cifra and Pospíšil, 2014)*.
3. Mitogenetic radiation has a specific temporal order, which gathers the signal.
  *This is based on experiments with “interrupted induction” (see Section The experimental setup). From them the authors concluded, that the “mitogenetic signal” from some inductors (microbial cultures, nerves, muscles, etc.) was a series of very short pulses (*~*10*^−3^ *s) coming at the frequency of* ~*10*^1^–*10*^2^ *Hz. On the contrary, radiation of malignant tumors was concluded continuous (Gurwitsch and Gurwitsch, 1934)*.
  *These conclusions are quite similar to the presently known data: UPE is indeed coming in short pulses (duration* <*10*^−3^ *s) and might have specific temporal order, that can be estimated with correlation, Fourier or wavelet analysis of the UPE signal (Kobayashi and Inaba, 2000; Beloussov et al., 2002). Yet whether it functions as a biological signal remains unknown*.

### Popp

Probably no other serious considerations were published on this question until 1980-s. The “early authors” were mainly involved in debates around the very existence of UPE and/or MGE (see discussion in Rahn, 1934a,b). The “UPE-ROS” groups (Tarusov, Chance, and others) considered UPE nothing but a side effect of destructive processes. Thus, the very question of UPE signaling was senseless for them. Other groups were focused on experimental work (Konev, Quickenden and others) or methods of UPE detection (Inaba's group).

The question of UPE signaling (i.e., mechanisms of distant communication of biological systems) was “revisited” by Popp. His main hypothesis was that biological systems possessed an inner coherent electromagnetic field, which generated photons in either coherent or the so-called squeezed quantum state. Thus, they could be easily detected by other coherent-state systems at practically any chaotic background (Popp, 2003).

Unfortunately, these beautiful ideas have not got experimental proof. There is no evidence of either coherent fields in biological systems or any coherent properties of UPE from them. There are also theoretical considerations that the longest possible coherent time for UPE from biological systems should not exceed 10^−9^ s (Mayburov and Volodyaev, 2009).

### Criticism

A serious critical work (Kucera and Cifra, 2013) published lately, considers physical limitations for UPE signaling, coming from the theory of information. Here are its main points.

According to Shenonn's theorem, the maximal capacity *C* of any communication channel in the presence of noise is $C=B\;\log_{2}\left(1+\frac{S}{N}\right)$, where *B* is the bandwidth of the channel (in *Hz*), *S*, intensity of the signal, *N*, intensity of the noise. The authors estimate these parameters from the following considerations:

1. *B* should not be too wide, because:
  - Propagation of electromagnetic field in the medium depends on its wavelength. Thus, a UPE signal composed of very different frequencies is inevitably distorted “on the way.”
  - Electromagnetic fields of different frequencies are generated through totally different mechanisms. Thus, it is very unlikely to have them working “in tune” in the same signaling system.
2. The wavelengths possibly used for signaling should be limited as follows:
  - No radiowaves, because there is no well-established mechanism for their reception.
    *That is right. Yet, there are a number of theoretical works (Binhi and Rubin, 2007), showing that non-equilibrium systems can have certain degrees of freedom with practically no energy exchange with the others. Thus, their excitation time can be very long, and they can work as “accumulators” of extremely weak EMFs*.
  - No infrared (IR), because thermal emission maximum from living systems lies in IR; hence it is very unlikely to have this region used for signaling.
    *This is true, but probably not enough to put a ban on this region. There is a large series of experimental works by Albrecht-Buehler (e.g., Albrecht-Buehler, 2005) showing IR sensing and IR interaction of cells. At least these results have to be thoroughly criticized (or explained by other means) before making such a conclusion*.
  - No UV, because “longer exposure to short UV is lethal.”
    *Dangerous doses of UV are* >*10*^6^ *times higher than intensity of UPE shown by Konev and Quickenden, and supposed intensity of mitogenetic radiation. Thus, this is not related to the topic*.
  - Only visible light, because there are no established mechanisms for generation and perception of EMF outside this region.
    *This is true concerning the widely appreciated mechanisms. Yet, generation and perception of EMF outside this region is not physically forbidden. Besides, aside from the whole MGE literature, there are serious works showing UV and IR emission by biological systems (Troitskii et al., 1961; Tilbury, 1992; Albrecht-Buehler, 2005)*.
3. The known UPE intensity is 10^0^–10^3^ photons/cm^2^/s.
4. The background intensity is up to 10^15^ photons/cm^2^/s.
5. Any kinds of signal filtering, like space or time filtering, phase sensing etc. require complicated machinery and hence are very unlikely for single cells.
  All this is true, yet not so simple. In particular:
  - *The background of 10*^15^ *photons/s/cm*^2^ *is full sunshine in visible spectral range. As MGE can be observed only in “semidarkness” (see Section The Experimental Setup), the estimated background should be* ~*10*^10^–*10*^12^ *photons/cm*^2^*/s in the visible range and 3*–*6 orders less in the UV*.
  - *All the measurements of spontaneous UPE are (naturally) performed in complete darkness. It is well known, that photon emission of any object taken from light, is initially* ~*10*^2^–*10*^3^ *more intense than its spontaneous UPE, and slowly decays in several hours (the phenomena of delayed luminescence and photo-induced chemiluminescence). Thus, optical levels in any biological system at external light are additionally excited, which can enhance the UPE intensity in real conditions by 1*–*2 orders of magnitude (Mayburov and Volodyaev, 2009)*.
    *As MGE is not observed at complete darkness, but only at lighter conditions, this additional excitation might be crucial for UPE signaling, and the real signal intensity can be 10*^1^–*10*^2^ *times higher, than presently supposed)*.
  - *A possible way to increase effective S/N ratio at given conditions is to transfer the signal as a series of short pulses with long intervals between them. This can increase the S/N ratio by a factor* $\alpha ={\left(\frac{t}{T}\right)}^{n}$, *where t, is duration of a pulse, T, interval between pulses, n, number of pulses encoding a single bit in the signal. Yet, this requires mutual “tuning” of interacting systems, which means history-dependence of signaling. This is not unfeasible, but rather complicated to perform (see Mayburov and Volodyaev, 2009 for more discussion)*.

The authors also point out that “no reported experiment <on distant interaction> shows absolute chemical separation <of the interacting objects>.” Hence, results of most of these works “should be attributed to another phenomenon.”

*This is true for many works in this area. Yet, a number of “early works” establish full chemical separation between the inductor and the recipient, examined with the isotope method (Gurwitsch and Gurwitsch, 1934). Besides, a number of works with no chemical separation of the interacting objects, use fully identical “chemical conditions” for experiment and control, with the only difference lying in transparency of the separating screen (Fels, 2009; Budagovskii et al., 2001)*.

Thus, we consider the authors' conclusion that “cellular signaling through light is either a paradox, or not accomplishable under natural conditions” unreasonably radical. Yet, main limitations for light signaling (if it exists) stated in the article are definitely correct and should be always accounted:

1. It cannot be observed at full daylight.
2. It cannot be observed at high distances.
3. It cannot transfer long “messages,” because they require either long time or high signal intensity. Hence, UPE signaling can only function as a trigger for previously prepared processes.
4. It should utilize spectral range with possibly lower background (the best “candidate” from this viewpoint is UV).

## Imitation of MGE with artificial sources of UPE

A number of authors tried to “simulate” MGE with artificial sources of UV. In Nakaidzumi and Schreiber (1931), Kreuchen and Bateman (1932), Richards and Taylor (1932), Seyfert (1932), Hollaender and Claus (1937) results were totally negative. Yet, methods used in these works were principally different from what had been recommended to detect MGE (see Sub-section Critical Works in Section MGE on Yeast and Bacteria). Thus, their negative results cannot be regarded serious or representative.

Stimulation of cell division with artificial ultraweak UV (on the objects, used as MGE recipients) was reported in Chariton et al. (1930), Ruyssen (1933). In Chariton et al. (1930) the most systematic results were published, with clear spectra of mitogenetic sensitivity of the recipients. The authors note, that stimulation effect in these experiments was obtained at much higher intensities than the estimates for radiation from biological MGE inductors. Hence, they suggest that it was not just intensity of UPE important for producing biological effect, but other parameters like temporal order, combination of spectral bands etc.

Very interesting data on this topic are mentioned in Gurwitsch and Gurwitsch (1934). Radiation from an arc lamp was weakened (details not mentioned), and its 254 nm spectral band was isolated and used as an artificial inductor. Standard induction of an “approved” recipient (yeast culture) gave no effect at any duration. Yet, “interrupted induction” (see Section The Experimental Setup) with single “induction pulses” of 0.7 ms and periodicity of 25 Hz gave definite MGE. Unfortunately, we couldn't find the original article or the “raw data.”

Later, Quickenden and Tilbury also tried to stimulate mitosis in yeast cultures with ultraweak UV (Quickenden et al., 1989). The above comparison of their conditions with earlier works remains true. Besides, the authors mentioned the presence of day light as a necessary condition for MGE in early works, but preferred to violate this recommendation on the basis of a personal opinion: “Celan et al. (1986) found that they [“early workers”] could only detect the mitogenetic effect in its [day light] absence.” Although scholastic conclusions are sometimes very trustworthy, they cannot be used as an argument against experiment.

## Discussion

Mitogenetic effect is presently what Wainwright called “forgotten microbiology” (Wainwright, 2000). Yet, as we intended to show, the literature on this topic is not just a number of non-scientific papers by “a few east-European workers.” It is an extensive research, performed in a dozen of countries by more than 150 authors, including very respectable scientists, and publications in highest rating journals. Where is this science now? Was it proven false since then?

As we tried to show in an evidence-based way, there are no serious works disproving MGE. The “common opinion” that MGE is “pathological science” (Hall, 1989; “Pathological Science”^2^) is a personal belief of a few influential scientists from the past (Bateman, 1935; Anonim, 1937; Hollaender and Claus, 1937), without any factual data, or with its very doubtful interpretation.

### False positive works – discrediting the topic

One cannot but agree that a number of works “confirming” MGE cannot be accounted as serious. As Hollaender wrote, “It is doubly unfortunate that the problem has attracted some workers who, apparently, see in the problem only an opportunity to deal in the spectacular” (Hollaender, 1936). A number of phenomena initially attributed to MGE were later shown to be either artifacts or of purely chemical origin [e.g., quorum sensing factors (Hogan, 2006; Shank and Kolter, 2009), CO_2_ (Hall et al., 2010; Volodyaev et al., 2013) or NH_3_-mediated interaction (Palková et al., 1997)].

Besides, in the present “open information” world the word “mitogenetic” is frequently used by people aside from science, what certainly discredits the topic in common opinion.

### Influential negative works

At the same time a number of “careful but negative” results, obtained by “a few Western authors” (Quickenden and Tilbury, 1985) came out strong against the phenomenon. Most of them were reviewed here in detail, to show principal methodical points, which prevented them from observing MGE. As Rahn wrote, “several investigators have claimed that their negative results disprove the positive results of others. That is a fallacy. When two investigators obtain different results, it does not prove that one has been right and the other wrong, it proves only that they didn't make the same experiment, that somewhere the conditions were different” (Rahn, 1934b).

There were also a number of very critical reviews clearly preconceived *pro* any (even badly done) negative works and *contra* any positive results of other workers. Thus, a review by Bateman (Bateman, 1935) was mostly based on finding disagreement between different “positive” works on MGE and debating with Gurwitsch's reasoning. Somehow the author mentioned only discrediting “positive” works, skipped any serious investigations (Acs, 1932; Frank, 1932; Tuthill and Rahn, 1933; Wolf and Ras, 1933), and regarded all “negative” works (discussed above) as the final disproof. An influential note in Nature (Anonim, 1937) totally based on (Hollaender and Claus, 1937), is another example of non-objective attitude, forming opinion.

Quickenden et al. are also quite critical in their reviews, but mix different areas. The three works they are always quoting as the final disproof are (Lorentz, 1929; Grey and Ouellet, 1933; Hollaender and Claus, 1937). Of them (Lorentz, 1929; Grey and Ouellet, 1933) were devoted to physical registration of UPE and had no biological experiments done. Thus, their negative results were utterly due to low sensitivity of their technique. The third work (Hollaender and Claus, 1937) was described above in detail.

### Historical and geopolitical factors

Of the “early works,” the most influential critical publications appeared right before WWII, and happened to be the “aftertaste” of this topic for the postwar science. Although a number of very good “positive” works appeared simultaneously, they were too late to gain traction before the War, and too early to be remembered after it. It can only be explained by this misfortune that a full appreciation of Audubert's work by Norrish (the Chair of Physical Chemistry at Cambridge University) and Vavilov (the President of The USSR Academy of Sciences at that time), were not a passport for a new wave of general research.

The post-war world was soon bound up in molecular biology. A few groups that were interested in photon emission, were either unnoticed (the groups of Anna Gurwitsch and Konev) or made their own revelations and shifted to other topics (Tarusov, Vladimirov, Zhuravlev).

This oblivion was redoubled by the language barrier and the Cold war, which kept Soviet and Western science apart. We have mentioned a number of important works by soviet biophysicists, which are mostly unknown even now.

### Uncertainty of the effect and its physiological role

Difficulties in achieving stable MGE are surely the most important negative factor for its acceptance. If MGE is so universal, why is it so difficult to “catch,” requiring so many peculiar actions? This is a standard question and a frequent “argument” against MGE. Although obviously no speculation is an evidence in experimental science, this point is really confusing.

Yet, in our opinion, the uncertainty of MGE is its natural and understandable property. Indeed, nearly every object used in MGE experiments consists of a huge number of cells. Characteristic distance between cells in culture is 10^−6^–10^−4^ m. Any external inductor is never closer to the recipient than 10^−3^–10^−2^ m, and separated with a few reflecting interfaces (quartz-air, quartz-liquid etc.). Thus, to get a detectable MGE, a worker needs to prepare the following conditions:

1. The inductor culture must be “strong” enough to produce MGE at such unnatural distances.
2. The recipient cells must be sensitive to external induction.
3. The internal MGE in the recipient culture should be possibly suppressed (but without damage).
4. The (extremely weak) MGE signal must be “preserved” from external disturbances “on its way” to the recipient.

Following these plain demands, one can easily understand the comprehensive conditions needed for MGE (see Section Methods of Observing MGE), including:

1. Careful selection of inductors and minimization of the distance inductor–recipient;
2. Still more careful preparation of recipients, including special phases of the culture itself and its “maternal culture” (the one it was seeded from);
3. Creating special, and even unnatural conditions for the recipient, including lower temperature, weakened medium, low culture density, etc., in such a way to get it “wakened,” but not defective;
4. Special precautions, like lower external light, no UV, very thin layers of the recipient culture, quartz of excellent purity, etc.

Looking back, it seems much more surprising that the MGE could ever be observed, than that it turned out so capricious.

### The present day paradoxical state-of-art…

**Figure 4:**
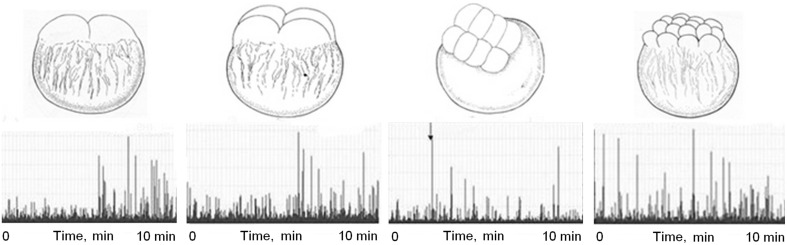
**“Fast dynamics” of UPE from fish eggs (***Misgurnus fossilis***, stages of 2, 4, 8, and 16 blastomers, morphology shown in the upper raw)**. Horizontal axis, time, min. Vertical axis, intensity of UPE per 0.1 s. Two successive emission bursts accompanying the 4th cleavage division (right frame) correlate with two waves of cell division. From Beloussov et al. (2000).

**Figure 5:**
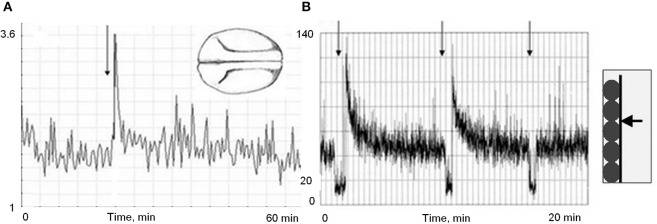
**Stress-induced photon emission, corresponding to “degradational radiation” (Gurwitsch and Gurwitsch, 1934)**. Horizontal axis, time, min; Vertical axis, intensity of UPE per 0.1 s. **(A)** An embryo of *Xenopus laevis* at the neurula stage (shown), abruptly cooled down from 23 to 4°C (marked with the arrow). From Volodyaev and Beloussov (2007). **(B)** A batch of *Misgurnus fossilis* eggs, gently pressed by a glass plate three times (vertical arrows, see scheme on the right) (from Beloussov, 2006). The first two pressure impulses caused pronounced emission bursts; the third one produced a minor response. This “depletion of degradational radiation” was also described in Gurwitsch and Gurwitsch (1934).

**Figure 6:**
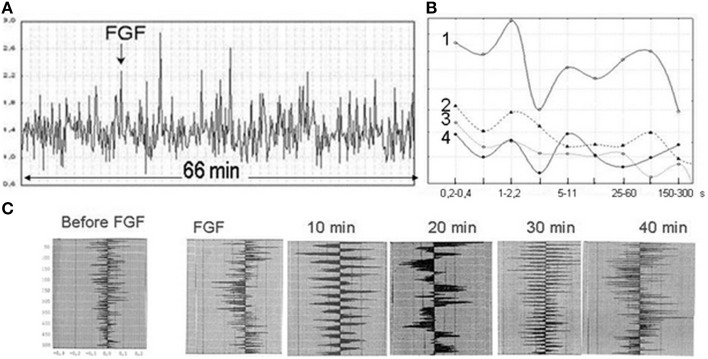
**UPE of a fibroblast culture (monolayer) after addition of a mitogenic agent (fibroblast growth factor FGF-1, 0.05 mg/ml)**. From Beloussov (2006). **(A)** UPE “fast dynamics” (FGF addition marked with the arrow). Horizontal axis, time, min; Vertical axis, intensity of UPE per 0.1 s. **(B)** Fourier spectra of UPE. Horizontal axis, period of the Fourier band (sec). Vertical axis, spectral density (log scale). Each plot is the average of five spectra (from independent measurements). (1) FGF-affected fibroblast culture; (2) intact cardyomyocyte culture; (3) cell-free medium; (4) intact fibroblast culture. Note similarity of the shape of (1) and (4), while spectral density of (1) is greatly enhanced. **(C)** A series of autocorellograms of Fourier spectra covering successive 10 min periods: before FGF-1 administration and 0–40 min after it. Note extensive temporal dynamics of photon emission.

**Figure 7:**
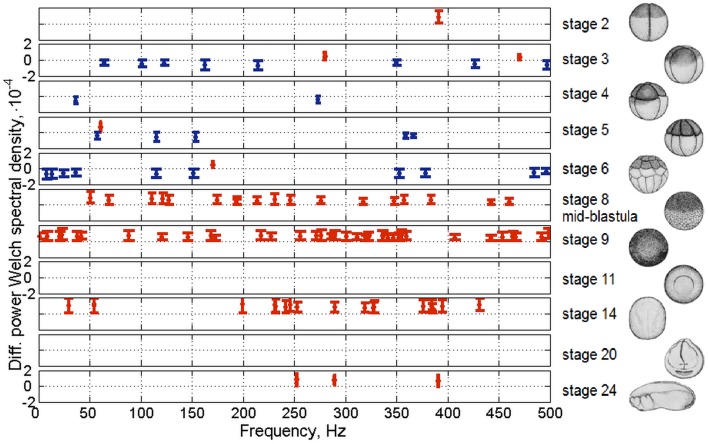
**Statistical Fourier spectra of UPE of single frog embryos ***(Xenopus laevis)*****. From Beloussov and Volodyaev (2013). Rows, different stages of development. Horizontal axes, frequency of the Fourier band (Hz); vertical axes, difference between mean spectral band of UPE and mean spectral band of the background noise (UPE from cuvette with medium). Shown only Fourier bands of UPE from embryos, significantly different from the background (*P* < 0.001). Whiskers, 99.9% confident intervals. Whiskers above zero (red)—Fourier band in UPE higher than in background, whiskers below zero (blue)—Fourier band in UPE lower than in background (do not mix with intensity of UPE). Stage numbers and morphology shown on the right. Stage 8, mid-blastula (known for abrupt activation of the embryo genome).

Let us take the most skeptical position and join those who claim that the basic mitogenetic effect does not exist at all. Then why were so many conclusions based exclusively on the biological detection of the “non-existing” phenomenon confirmed with physical methods later? Let us briefly enumerate the main ones:

- Photon emission from onion roots, cleaving eggs, early chicken embryos, budding yeast, excited nerves, working heart, malignant tumors and several chemical reactions, first discovered with the use of biological “detectors,” was later confirmed by physical measurements (Quickenden and Que Hee, 1974; Tilbury, 1992; Beloussov et al., 1997, 2000, 2002; Beloussov, 2002; Volodyaev and Beloussov, 2007) (see Figures 4–6).
- The phenomenon of “degradational radiation” (see Section MGE as a Non-invasive Probe for Detecting Physiological and Pathological States of Cells and Tissues) was also reproduced by physical methods (Beloussov, 2002; Volodyaev and Beloussov, 2007) (see Figure 5).
- The predicted spectral range of mitogenetic radiation was confirmed by physical devises (Troitskii et al., 1961; Gurwitsch et al., 1965; Quickenden and Que Hee, 1976; Quickenden and Tilbury, 1991; Tilbury and Quickenden, 1992).
- Already in 1930-s mitogenetic radiation was concluded to be of intermittent character and consist of millisecond flashes. Recently this was confirmed by direct observations (Beloussov and Volodyaev, 2013) (see Section Can UPE Transfer Information? and Figures 4–7).
- A personal reminiscence of one of the authors (LB): “When studying UPE from chicken embryos in Prof. Popp's lab (with the use of PMT), I found significant UPE in UV range up to the 2nd day of incubation (Beloussov et al., 1997). As I recognized later, just the same emission period was detected with the use of the onion root “detector” as far as in 1920-s.”

What might be even more important than these particular coincidences, are the above-mentioned generalizations. The presently well-established role of excited and non-equilibrium states of biomolecules and their complexes, was claimed by the “mitogenetic school” long before any other references. Taking into account that at least some of these data (first of all the data on tumor pathology) are not only of academic, but also of applicatory values, it would be, by the authors' opinion, unpardonable to leave apart this direction of research as false. Rather, we are dealing with situation which may be compared with reaching an unknown land in a small boat without a reliable navigation and hence without a certainty that the land can be reached at any next attempt. Nevertheless, the land is likely to exist, and the navigation is considerably improved. Thus, let us try to outline a kind of road map for the further work in this field.

### …And a road map for the near future

If someone decides to address this topic again, they should consider a few important points.

1. MGE cannot be detected off the cuff. There are plenty of pitfalls on the way, and even a definitely positive try can fail to be reproduced. One has to take into account both positive and negative experience of other workers, and study thoroughly all methodical details and precautions.
  Even this might not be enough. As Rahn writes, “It must be stated with perfect frankness that biological detectors sometimes fail for unknown reasons…These occasional failures have nothing to do with the error of the method. When mitogenetic effects are observed, they are outside the limits of error” (Rahn, 1936).
  We strongly recommend careful inquiry of methodical recommendations in Rahn (1936), Gurwitsch and Gurwitsch (1945) for any research in this topic.
2. In general, investigating biological processes by means of their external stimulation is quite a difficult and dubious way. A much more powerful tool of the present day research is inhibitory analysis (in broad sense). This approach is likely to be more effective than numerous attempts to stimulate cell division. Thus, it should (and can be) also applied to this area. At present time, a large set of agents inhibiting or screening radiation are available and their usage can elucidate a disputable question on the biological role of endogenous photon emission.
  In our opinion, a promising way of working with MGE would be to suppress internal mitogenetic stimulation in the system, and then stimulate cell division in it by external inductors of MGE. It is certainly complicated to figure out an agent, specifically suppressing MGE without any toxic effect. But first, there is some information available on this topic (Gurwitsch and Gurwitsch, 1959; Gurwitsch, 1968), and second, it might be the most vivid method of addressing the effect.
3. Next, it might be reasonable to temporally leave apart the biological effect and concentrate on physical measuring devices, which have been greatly improved since Gurwitsch's times. This means also that we have to abandon, at least for some time, detailed spectral analysis of the radiation, which was based almost exclusively on “biological detectors.” What may be suggested instead, is analysis of frequency spectra (Fourier or wavelet), which may be obtained with modern technique within a wide range of characteristic times. As shown by tentative experiments (Beloussov, 2002, 2006; Volodyaev and Beloussov, 2007), such analysis reveals a definite radiation component in the responses of cells to the action of various non-specific stresses (Figure 5), cytoskeletal inhibitors and growth factors (Figure 6). The latter is of a special interest, because it confirms connections between photon emission and induction of cell division (performed in these experiments by chemical agents).
  In addition, Fourier analysis permitted to observe the radiation component in the crucial developmental event, known as mid-blastula transition and associated with the burst of embryonic genes activation (Figure 7). In any case, detection of the optical range energies obeying regular temporal patterns greatly enriches our view upon cell signaling, gene expression and the function of cytoskeletal components.
4. It is also unpardonable to ignore mitogenetic data on cancer pathologies. We must definitely know whether the “cancer quencher” claimed by a number of authors, really exists in blood of cancer patients, and what role in the development of the disease it might play.
5. And the last, but definitely not the least. Physical measure of UPE cannot give any information about its biological role (if there is any). A fully parallel research of biological MGE and UPE from the same objects, and most important, under identical conditions (including temperature, light, aeration, etc…) should be done, if a good and stable biological effect is obtained. This could be a breakthrough for understanding the mechanisms of both sending and receiving the “MGE signal.”

The authors' view is that research on MGE should be renewed with the use of the entire set of powerful approaches acquired by modern science.

### Conflict of interest statement

The authors declare that the research was conducted in the absence of any commercial or financial relationships that could be construed as a potential conflict of interest.

## Footnotes

^1^The post-diauxic growth phase starts after the growth curve bend point (diauxic shift) and lasts for 5–10 days (Herman, 2002; Gray et al., 2004). It's characterized with slow growth, oxygen-based metabolism and slow reorganization toward the stationary phase. The real stationary phase (G_0_) starts after that and is much closer to dormitant states. These two states were mixed until 1990-s. Rahn used cultures 1–6 days old (grown at 30°C), without buds, which they called stationary (Tuthill and Rahn, 1933; Rahn, 1936). These cultures are definitely not G_0_ (which starts 7–10 days after inoculation at these conditions), and according to the present-day terminology are post-diauxic. As this phase is transitional (from growth to stationary state), the exact age of the culture can also be important for its sensitivity to MGE (see below).

^2^“Pathological Science.” http://en.wikipedia.org/wiki/Pathological_science#cite_note-9.
